# CD44 expression positively correlates with Foxp3 expression and suppressive function of CD4^+ ^T_reg _cells

**DOI:** 10.1186/1745-6150-4-40

**Published:** 2009-10-23

**Authors:** Tie Liu, Lynn Soong, Gang Liu, Rolf König, Ashok K Chopra

**Affiliations:** 1 Department of Microbiology and Immunology, University of Texas Medical Branch, Galveston, TX, USA; 2 Department of Medicine, Feinberg School of Medicine, Northwestern University, Chicago, IL, USA

## Abstract

**Background:**

CD4^+^CD25^+ ^regulatory T (T_reg_) cells develop in the thymus and can suppress T cell proliferation, modulated by Foxp3 and cytokines; however, the relevance of CD44 in T_reg _cell development is less clear. To address this issue, we analyzed Foxp3 expression in CD44^+ ^T_reg _cells by using multiple parameters, measured the levels of the immunoregulatory cytokine interleukin (IL)-10 in various thymocyte subsets, and determined the suppressor activity in different splenic T_reg _cell populations.

**Results:**

Within mouse thymocytes, we detected T_reg _cells with two novel phenotypes, namely the CD4^+^CD8^-^CD25^+^CD44^+ ^and CD4^+^CD8^-^CD25^+^CD44^- ^staining features. Additional multi-parameter analyses at the single-cell and molecular levels suggested to us that CD44 expression positively correlated with Foxp3 expression in thymocytes, the production of IL-10, and T_reg _activity in splenic CD4^+^CD25^+ ^T cells. This suppressive effect of T_reg _cells on T cell proliferation could be blocked by using anti-IL-10 neutralizing antibodies. In addition, CD4^+^CD25^+^CD44^+ ^T_reg _cells expressed higher levels of IL-10 and were more potent in suppressing effector T cell proliferation than were CD4^+^CD25^+^CD44^- ^cells.

**Conclusion:**

This study indicates the presence of two novel phenotypes of T_reg _cells in the thymus, the functional relevance of CD44 in defining T_reg _cell subsets, and the role of both IL-10 and Foxp3 in modulating the function of T_reg _cells.

**Reviewers:**

This article was reviewed by Dr. M. Lenardo, Dr. L. Klein & G. Wirnsberger (nominated by Dr. JC Zungia-Pfluker), and Dr. E.M. Shevach.

## Background

T_reg _cells are important in the control of self-reactive T cells, contributing to the maintenance of immunological self-tolerance [1]. T_reg _cells develop in the thymus through a process involving the recognition of self-peptides presented by major histocompatibility complex (MHC) molecules and driven by high-affinity interactions between the T cell receptor (TCR) on developing thymocytes and self peptide-MHC complexes on thymic epithelial cells [2-5]. Forkhead box P3 (Foxp3), an X-chromosome-linked factor that controls T_reg _cell development and function, is generally thought to also control positively the functions of T_reg _cells in a binary fashion, as Foxp3 expression is sufficient to specify immune-suppressive activities in conventional T cells [6]. Although Foxp3 is considered as a specific marker for the T_reg _cell lineage [7,8], its expression pattern during thymocyte development remains less clear.

T_reg_-mediated suppression is cell contact dependent [9], but the immunosuppressive cytokines transforming growth factor (TGF)-β and IL-10 also play an important role [10-12]. The collective activity of TGF-β and IL-10 ensures a controlled inflammatory response specifically targeting pathogens without evoking excessive immunopathology to self-tissues [13]. IL-10 is a cytokine which is an essential molecule in the mechanism underlying suppression mediated by T_reg _cells. It has anti-inflammatory activity and indirectly suppresses cytokine production and proliferation of antigen-specific CD4^+ ^T effector cells. IL-10 is produced by subsets of CD4^+ ^cells with regulatory function [14]. More specifically, it has been shown that IL-10 produced by T_reg _cells is essential for *in vivo *suppression, as IL-10-deficient T_reg _cells can not regulate T cell induced colitis [15,16]. TGF-β and IL-10 are potent mediators of immune suppression, and T_reg _cells can not only use TGF-β and IL-10 to perform their immunosuppression function, but also to educate other CD4^+^CD25^-^cells to become T_reg _cells [12].

The adhesion molecule CD44 (synonymous with Pgp1, HUTCH-1, or ECM-III) is the principal receptor for hyaluronic acid. Multiple functions and cellular responses have been attributed to the activation of CD44, including the induction of cell motility, activation of cell survival responses, and promotion of cell adhesion [17]. Although CD44 has a wide tissue distribution, its expression during a particular stage or in a subset of thymocyte progenitors suggests a functional involvement of CD44 in homing and thymic colonization of precursor cells [18]. Although differential expression levels of CD44 among different subsets of thymocytes have been reported [19], its biological relevance in T cell differentiation is unclear.

In this study, we used naïve C57BL/6 mice and performed six-color flow cytometry and real-time reverse transcriptase (RT)-polymerase chain reaction (PCR) analyses, as well as *in vitro *T cell suppression assays. We present herein the following key findings: 1) the surface expression of CD44 in mouse thymocytes positively correlated with that of Foxp3; 2) CD4^+^CD25^+^CD44^+ ^T_reg _cells expressed higher levels of IL-10 and were more potent in suppressing effector T cell proliferation than were CD4^+^CD25^+^CD44^- ^cells; and 3) blocking IL-10 aborogated suppressive mechanisms of CD4 T_reg _cells. Our data suggest that T_reg _cells could be further divided into three subsets based on CD44 expression levels, with CD4^+^CD25^+^CD44^high ^cells displaying the highest levels of IL-10 production and having regulatory functions.

## Methods

### Mice

Female C57BL/6 mice (Taconic Farms, Germantown, NY) were maintained under specific pathogen-free conditions, and used for experimentation at 4 to 6 weeks of age, according to protocols approved by the UTMB Institutional Animal Care and Use Committee and NIH guidelines.

### Flow cytometric analysis

Thymocytes and splenocytes were obtained from naïve mice and suspended in phosphate-buffered saline (PBS) and 1% fetal calf serum (FCS). To avert non-specific binding to mouse Fcγ receptors, cells were blocked with mouse CD16/CD32 mAb (0.25 μg/100 μl) (BD Biosciences, Franklin Lakes, NJ) for 15 min. After washing, cells were stained for the expression of CD4 (PE-Cy7, clone RM 4-5), CD8α (FITC, clone 53-6.7), CD44 (PE, clone 1M7), CD25 (APC-Alexa Fluor755, clone PC-61.5), or TCR-β (PerCp, clone H57-597) at 4°C for 60 min. In some cases, the surface-stained cells were fixed/permeabilized with a Cytofix/Cytoperm kit (BD Biosciences) and then stained with Foxp3 (PE-Cy5, clone FJK-16s) or IL-10 (PerCP, clone JESS-16E3) at 4°C for 45 min. The corresponding isotype controls (rat IgG1, IgG2a, and IgG2b) were purchased from eBioscience (San Diego, CA) and BD Bioscience, respectively. Cells were analyzed using a FACScan (BD Biosciences) and BD FACSDiva software (BD Biosciences).

### Isolation of CD4^+ ^T subsets

Splenocytes were collected from C57BL/6 mice, treated with RBC lysis buffer (Sigma, St. Louis, MO), and T cells were enriched by passage through nylon-wool columns and subsequently purified. Briefly, splenocytes (10^8^) were incubated in the column at 37°C in the presence of 5% CO_2 _for 1 h before eluting the cells with RPMI medium. Cells were then stained for the surface expression of CD4, CD8, CD44, and CD25 by incubating with the appropriate antibodies at 4°C for 60 min. After washing, the following subsets of CD4^+ ^T cells were isolated by using a FACSAria (BD Biosciences): CD4^+^CD25^+^CD44^+^, CD4^+^CD25^+^CD44^-^, CD4^+^CD25^+^CD44^high^, CD4^+^CD25^+^CD44^med^, and CD4^+^CD25^+^CD44^low ^cells. Purified T cell subsets were immediately used for subsequent analyses.

### RT-PCR and real-time RT-PCR

Total RNA was extracted from purified T cell subsets. The first-strand cDNA was synthesized from 2 μg of RNA using reverse transcriptase (SuperScript III, Invitrogen). An aliquot of first-strand cDNA was amplified by Ampli-*Taq *(Perkin-Elmer Cetus, Norwalk, CT) in a total volume of 50 μl reaction buffer consisting of 10 mM Tris-HCl (pH 8.3), 50 mM KCl, 1.5 mM MgCl_2_, 0.001% gelatin, and 0.2 mM deoxynucleoside triphosphate. The primers for RT-PCR were: IL-10 forward 5'-CAGACTCTTAAACACCGAGCCG-3', reverse 5'-GACTTAGCAAGACACGATGCGA-3'; and β-actin forward 5'-TGGAATCCTGTGGCATCCATGAAAC-3', reverse 5'-TAAAACGCAGCTCAGTAACAGTCCG-3'. The PCR reaction included one cycle of initial amplification (at 94°C for 5 min, 56°C for 3 min, 72°C for 2 min), followed by 22 to 32 cycles at 94°C for 1 min, annealing at 56°C for 1 min, and extension at 72°C for 0.5 min. The final extension reaction was prolonged to 10 min at 72°C. After amplification, PCR products were separated by electrophoresis through 1-2% agarose gels.

Quantitative RT-PCR was performed at the Real-Time PCR Core Facility, Sealy Center for Cancer Cell Biology, UTMB. We used Applied Biosystems (Foster City, CA) assay-by-design and assay-on-demand 20× assay mixes of primers and TaqMan MGB probes (FAM™ dye-labeled) for our target genes (IL-10) and a pre-developed 18S rRNA (VIC™-dye labled probe) TaqMan^® ^assay reagent for an endogenous control. Real-time RT-PCR was performed with 40 ng cDNA, using a universal PCR master mix reagent kit (Applied Biosystems) and the following cycling parameters: Uracil-N-glycosylase (UNG) activation at 50°C for 2 min, AmpliTaq activation at 95°C for 10 min, denaturation at 95°C for 15 sec and annealing/extension at 60°C for 1 min (repeat 40 times) on ABI7000. Duplicate C_T _values were analyzed *via *an in Microsoft Excel program using the comparative C_T _(ΔΔC_T_) method, as described by the manufacturer (Applied Biosystems). The amount of target (2^-ΔΔCT^) was obtained and normalized to the endogenous reference (18s) and relative to a calibrator (one of the experimental samples).

### *In vitro *T cell suppression assay

Splenic T cells were prepared by passing splenocytes through nylon-wool columns (10^8 ^splenocytes were incubated in the column at 37°C in the presence of 5% CO_2 _for 1 h before eluting the cells with RPMI medium). Effector T cells were pre-cultured with Concanavalin A (Con A, 20 μg/ml) for 2 h, washed, and then seeded in 96-well U-bottom microtiter plates (2 × 10^5^/well), to which T_reg _cells were added at T_reg_-to-effector cells in a ratio of 1:2. After cultivation in the presence of Con A (2 μg/ml) for 72 h, cells were pulsed with [^3^H]-thymidine for the last 10 h, and incorporation of radioactivity was measured by liquid scintillation counting in triplicate. Data were expressed as the arithmetic means ± standard deviation (S.D.).

To assess the involvement of IL-10 in reversing T cell suppression, we sorted CD4^+ ^CD25^- ^and CD4^+ ^CD25^+ ^CD44^+ ^(or CD4^+^CD25^+ ^CD44^-^) cells *via *FACS. T cells (in RPMI medium) were incubated alone or co-cultured with T_reg _cells at a 2:1 ratio with 1 μg/ml plate-bound anti-CD3 (145.2C11, BD Bioscience) in 96-well U-bottom plates. In some cases, blocking anti IL-10 antibodies (1B1.3a, 100 ng/ml, BD Bioscience) or rat IgG1 isotype control (R3-34, 100 ng/ml, BD Bioscience) were added, and the above mixture of cells were incubated at 37°C in the presence of 5% CO_2 _for 72 h. Subsequently, cells were pulsed with [^3^H]-thymidine for the last 10 h, and incorporation of radioactivity was measured by liquid scintillation counting in triplicate. Data were expressed as the arithmetic means ± S.D.

### Statistical analysis

At least three independent experiments were performed, and the difference between two groups was determined using Student's *t*-test. One- or two-way ANOVA was used for multiple group comparisons (GraphPad Software v 4.0, San Diego, CA). Statistically significant values were referred to as follows: *, *p *< 0.05; **, *p *< 0.01; ***, *p *< 0.001.

## Results

### Detection of CD4^+^CD8^-^CD25^+^CD44^- ^and CD4^+^CD8^-^CD25^+^CD44^+ ^T cells in the thymus

**Figure 1:**
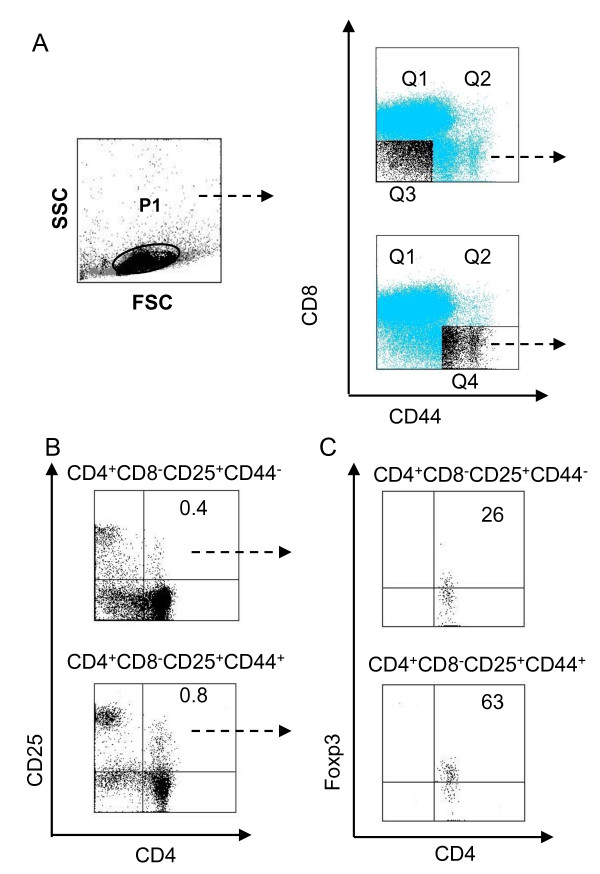
**Detection of CD4^+ ^CD8^- ^CD25^+ ^CD44^+ ^and CD4 ^+ ^CD8^- ^CD25^+ ^CD44^- ^T_reg _cells in the thymus**. **(A) **Cell gating strategy and staining patterns. Thymocytes were obtained from naïve C57BL/6 mice and stained with specific antibodies against CD4 (Cy7-PE), CD8 (FITC), CD44 (PE), and CD25 (APC-Alexa Fluor755). Cells in P1 were gated as live thymocytes. **(B) **Based on CD44 and CD8 expression, CD4^+^CD8^-^CD25^+^CD44^+ ^and CD4^+^CD8^-^CD25^+^CD44^- ^cells were defined. **(C) **Foxp3 expression in CD4^+^CD8^-^CD25^+^CD44^+ ^and CD4^+^CD8^-^CD25^+^CD44^- ^cells. The data are shown as the percentage of total, live thymocytes (population P1) in each cell subset, and are presented as mean ± S.D. from four independent experiments.

CD4^+^CD25^+ ^cells are members of a unique lineage of T cells that are selected during the process of T cell development in the thymus; however, the location and sequence of T_reg _cell development remain unclear [20-22]. To address this issue, we first examined the profile of thymocytes in naïve mice using multi-color flow cytometry for the simultaneous detection of CD4, CD8, CD25, and CD44 cells. As shwon in Figure 1A, live thymocytes were gated as P1, and these thymocytes were subsequently divided into four populations based on CD44 and CD8 expression (identified by quadrants Q1-Q4), yielding the CD4^+^CD8^-^CD25^+^CD44^- ^population from Q3 and the CD4^+^CD8^-^CD25^+^CD44^+ ^population from Q4. These populations of CD4^+^CD8^-^CD25^+^CD44^- ^and CD4^+^CD8^-^CD25^+^CD44^+ ^single positive (SP) cells were comprised of only 0.4% and 0.8% of total thymocytes, respectively (Figure 1B). Furthermore, Foxp3 expression was detacted in 63% of CD4^+^CD8^-^CD25^+^CD44^+ ^cells and 26% of CD4^+^CD8^-^CD25^+^CD44^- ^cells (Figure 1C). These staining and gating approaches allowed us to detect 16 distinct subsets of cells.

### TCR-β expression in subsets of T_reg _cells in the thymus

**Figure 2:**
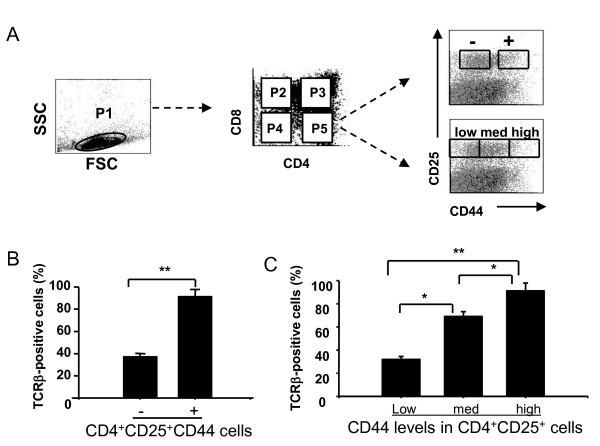
**TCR-β expression on CD4^+ ^CD8^- ^CD25^+ ^CD44^+ ^T _reg _cells in mouse thymus**. Thymocytes were collected from naïve mice, stained with antibodies against CD4 (PE-Cy7), CD8 (FITC), CD44 (PE), CD25 (APC-Alexa Fluor755), and TCR-β(PerCp), and analyzed by flow cytometry. **(A) **CD4^-^CD8^- ^(P4), CD4^+^CD8^+ ^(P3), and CD4^+^CD8^- ^cells (P5) were gated from total, live thymocytes (P1). Cells from each of the quadrants (designated as P3-P5) were subgated into CD25^+^CD44^- ^and CD25^+^CD44^+ ^(In Figure 2A, - and + mean CD25^+^CD44^- ^and CD25^+^CD44^+ ^cells [analysis described in panel B]). In addition, CD4^+^CD8^- ^SP cells were subgated into CD25^+^CD44^low^, CD25^+^CD44^med^, and CD25^+^CD44^high ^cells (analysis described in panel C). (**B) **The percentages of TCR-β cells in CD4^+^CD8^- ^SP cells (mean ± S.D., n = 3). **(C) **The percentages of TCR-β cells among CD4^+^CD25^+^CD44^low^, CD4^+^CD25^+^CD44^med^, and CD4^+^CD25^+^CD44^high ^cells. Statistical significance in panels B and C is indicated by * *p *< 0.05 and ** *p *< 0.01.

Since a productive TCR-β gene rearrangement is a critical event in thymocyte development and proliferation [23,24], we then examined TCR-β expression in different subsets of thymocytes. For Figure 2A, total live thymocytes (P1) were gated, as described for Figure 1. Based on expression of CD4 and CD8, we defined subpopulations of CD4^-^CD8^- ^as P4, CD4^+^CD8^+ ^as P3, and CD4^+^CD8^- ^cells as P5, respectively. Each of these subpopulations was further analyzed for expression of CD25 and CD44. Finally, for each of the resulting nine subpopulations, we measured TCR-β expression. We observed that CD4^+^CD8^-^CD25^+^CD44^+ ^cells contained a higher percentage of TCR-β^+ ^cells than did CD4^+^CD8^-^CD25^+^CD44^- ^cells (Figure 2B). To further define the role of CD44 expression in the development of CD4^+^CD25^+ ^cells, we subdivided CD4^+^CD8^-^CD25^+^CD44^+ ^SP cells into CD4^+^CD8^-^CD25^+^CD44^low^, CD4^+^CD8^-^CD25^+^CD44^med^, and CD4^+^CD8^-^CD25^+^CD44^high ^cells (Figure 2A). We then analyzed the percentage of TCR-β-expressing cells in each subpopulation (Figure 2C). Among CD4^+^CD8^-^CD25^+^cells, the CD44^high ^subpopulation expressed TCR-β at the highest frequency and intensity, whereas the CD44^low ^subpopulation displayed the lowest expression levels of TCR-β. Thus, the surface expression of CD44 was positively correlated with TCR-β expression, suggesting that the CD4^+^CD25^+ ^CD44^high ^cells represent a more mature subset of T cells.

### Foxp3 and CD44 expression in mouse thymocytes and splenocytes

**Figure 3:**
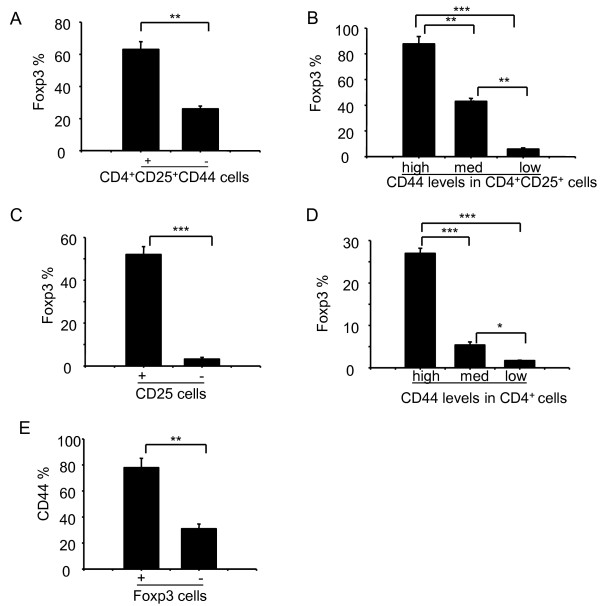
**Foxp3 expression in different subsets of thymocytes**. Thymocytes were stained with antibodies against CD4 (PE-Cy7), CD8 (FITC), CD44 (PE), CD25 (APC-Alexa Fluor755), and Foxp3 (PE-Cy5). CD4^-^CD8^- ^DN, CD4^+^CD8^- ^SP, CD4^-^CD8^+ ^SP, and CD4^+^CD8^+ ^DP thymocytes were gated for subsequent analyses. **(A) **Foxp3 expression (%) in CD4^+^CD25^+ ^cells that was either positive or negative for CD44. **(B) **Foxp3 expression (%) in CD4^+^CD25^+ ^cells that expressed high, medium, or low levels of CD44. **(C) **Foxp3 expression (%) in CD4^+ ^cells that was either positive or negative for CD25. **(D) **Foxp3 expression (%) in CD4^+ ^cells that expressed high, medium, or low levels of CD44. **(E) **CD44 expression (%) in CD4^+ ^cells that was either positive or negative for Foxp3. Panels A to E show representative results with mean ± S.D. from three independent experiments (* *p *< 0.05, ** *p *< 0.01, *** *p *< 0.001).

To test whether there are functional differences between different subsets of CD44-expressing cells, we examined the expression profile of Foxp3. Thymocytes derived from naïve mice were stained with antibodies against CD4, CD8, CD25, CD44, and Foxp3 (Figure 3). In three independently performed experiments, we consistently found that a significantly higher proportion of CD4^+^CD25^+^CD44^+ ^cells expressed Foxp3 as compared to CD4^+^CD25^+^CD44^- ^cells (63% vs. 26%, *p *< 0.01, Figure 3A). We also observed a positive correlation between CD44 and Foxp3 expression among CD4^+^CD25^+ ^cells. Foxp3 was detected in 87% of the CD4^+^CD25^+^CD44^high ^cells, 41% of the CD4^+^CD25^+^CD44^med ^cells, and 5.9% of the CD4^+^CD25^+^CD44^low ^cells (Figure 3B). Consistent with another report [2], a higher proportion of CD4^+^CD25^+^cells expressed Foxp3 compared to CD4^+^CD25^- ^cells (52% vs. 3.2%, *p *< 0.01, Figure 3C). Again, the CD4^+^CD44^high ^population contained the highest proportion of Foxp3-expressing cells (Figure 3D). Most of the CD4^+^Foxp3^+^cells also expressed CD44 (78%), whereas 31% of CD4^+^Foxp3^- ^cells expressed CD44 (Figure 3E). Thus, we found a positive correlation between Foxp3 and CD44 expression, suggesting that CD44 may be an additional marker for the maturation of regulatory thymocytes.

**Figure 4:**
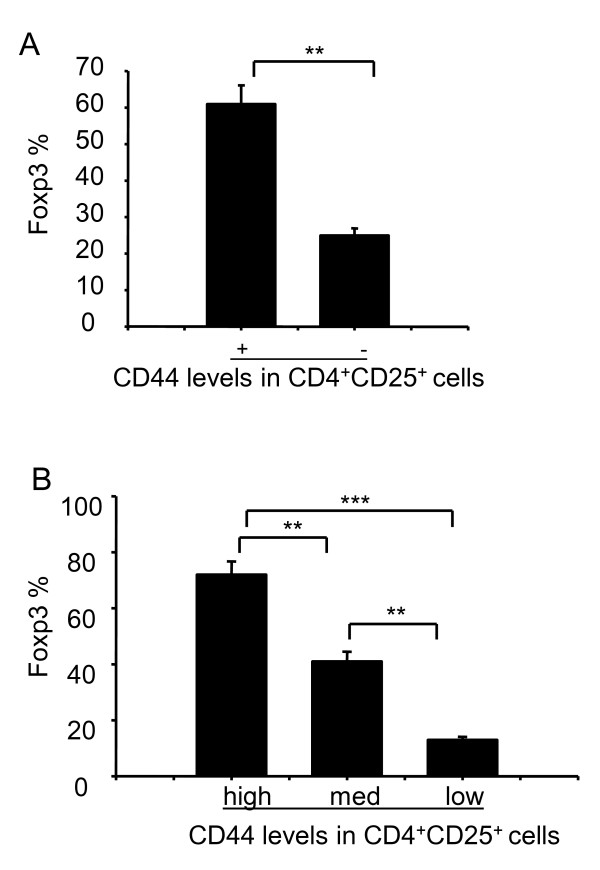
**Foxp3 expression in different subsets of splenocytes**. Splenocytes were stained with antibodies against CD4 (PE-Cy7), CD8 (FITC), CD44 (PE), CD25 (APC-Alexa Fluor755), Foxp3 (PE-Cy5) and analyzed by flow cytometry. **(A) **Foxp3 expression (%) in CD4^+^CD25^+ ^cells that was either positive or negative for CD44. **(B) **Foxp3 expression (%) in CD4^+^CD25^+ ^cells that expressed high, medium, or low levels of CD44. Shown are representative results with mean ± S.D. from three independent experiments (** *p *< 0.01, *** *p *< 0.001).

To validate these findings, we also stained splenocytes with antibodies against CD4, CD8, CD25, CD44, and Foxp3. We found a significantly higher proportion of CD4^+^CD25^+ ^CD44^+ ^cells expressed Foxp3 than that of CD4^+^CD25^+^CD44^- ^cells (61% vs. 25%, Figure 4A), suggesting a positive correlation between CD44 and Foxp3 expression among CD4^+^CD25^+ ^cells. Foxp3 was detected in 72% of the CD4^+^CD25^+^CD44^high ^cells, 42% of the CD4^+^CD25^+^CD44^med ^cells, and 13% of the CD4^+^CD25^+^CD44^low ^cells (Figure 4B).

### Functional assessment of T_reg _cell subsets in the thymus and spleen

**Figure 5:**
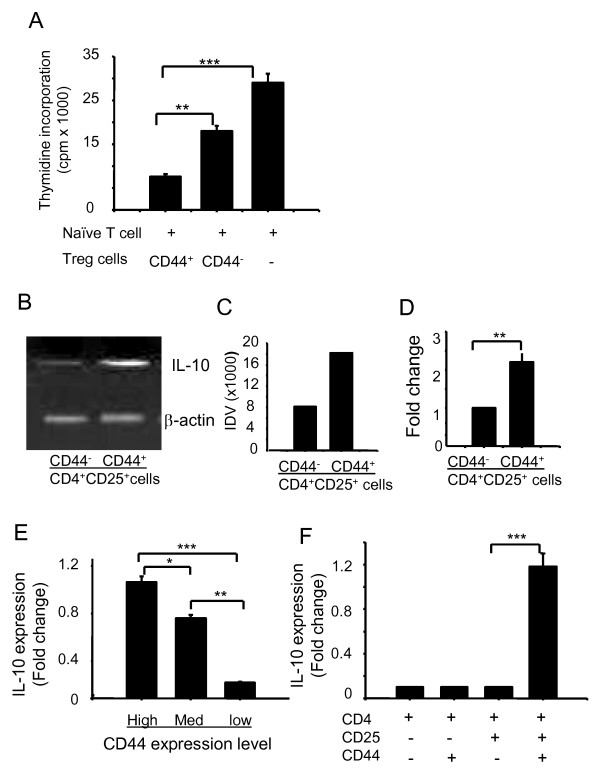
**Regulatory function of CD4^+ ^CD25^+ ^CD44^+ ^and CD4^+ ^CD25^+ ^CD44^- ^splenocytes**. **(A) **CD4^+^CD25^+^CD44^+ ^and CD4^+^CD25^+^CD44^-^cells were sorted from the splenocytes of C57BL/6 mice, and these cells (1 × 10^5^/well) were co-cultured with naïve T cells (2 × 10^5^/well) in the presence of Con A for 72 h. T cell proliferation was measured by incorporation of [^3^H]-thymidine. Data are presented as counts per min, and shown are the mean ± S.D. from three independent experiments. **(B) **Total RNA was extracted from purified CD4^+^CD25^+^CD44^+ ^and CD4^+^CD25^+^CD44^- ^cells for RT-PCR analysis of IL-10 expression. **(C) **The integrated density values (IDV) for the IL-10 transcripts were quantitated and normalized to those of β-actin. Shown are representative results from one of three independent experiments. **(D) **The data were pooled from three independent experiments and shown in the plot. **(E) **The levels of IL-10 mRNA among different subsets of CD44^+ ^expressing cells. CD4^+^CD25^+^CD44^high^, CD4^+^CD25^+^CD44^med ^and CD4^+^CD25^+^CD44^low ^cells were sorted from the spleens of naïve mice. **(F) **CD4^+^CD8^-^CD25^+^CD44^+^, CD4^+^CD8^-^CD25^+^CD44^-^, CD4^+^CD8^-^CD25^-^CD44^+^, and CD4^+^CD8^-^CD25^+^CD44^- ^cells were sorted from the thymus of naive mice. Total RNA was isolated for measuring IL-10 mRNA by real-time RT-PCR. Data are presented as fold-induction relative to the levels of β-actin. Shown are representative results one of three independent experiments.(* *p *< 0.05, ** *p *< 0.01, *** *p *< 0.001).

To examine whether CD4^+^CD25^+^CD44^+ ^and CD4^+^CD25^+^CD44^- ^cells differ in their ability to suppress effector T cells, we sorted CD4^+^CD25^+^CD44^+ ^and CD4^+^CD25^+^CD44^- ^cells from the spleen by FACS. Isolated cell populations were then co-cultured with naïve T cells pre-activated with Con A for 2 h. After co-culture of effector T cells (2 × 10^5^/well) with T_reg _cells in the presence of Con A (2 μg/ml) for 72 h, T cell proliferation was measured. As shown in Figure 5A, although CD4^+^CD25^+^CD44^+ ^and CD4^+^CD25^+^CD44^- ^cells were capable of suppressing T cell proliferation, CD4^+^CD25^+^CD44^+ ^cells were significantly more potent suppressors than were CD4^+^CD25^+^CD44^- ^cells. Because the expression of IL-10 is a hallmark of T_reg _cells [12,16], we examined *via *RT-PCR the levels of IL-10 mRNA in purified CD4^+^CD25^+^CD44^+ ^and CD4^+^CD25^+^CD44^- ^cells. The levels of IL-10 were 2 to 2.4-fold higher in CD4^+^CD25^+^CD44^+ ^cells than in CD4^+^CD25^+^CD44^- ^cells, as determined by densitometry scanning of the gels (Figure 5B and 5C) and the data were pooled from three independent experiments and shown in a plot (Figure 5D). To confirm these findings, we sorted CD4^+^CD25^+^CD44^high^, CD4^+^CD25^+^CD44^med^, and CD4^+^CD25^+^CD44^low ^cells from the spleens and measured the levels of IL-10 mRNA by real-time RT-PCR. As shown in Figure 5E, CD4^+^CD25^+ ^CD44^high ^T_reg _cells expressed 5-fold higher levels of IL-10 than did CD4^+^CD25^+^CD44^low ^cells. Thus, the CD44 expression levels and T_reg _function were positively correlated. To further examine the intrathymic development of T_reg _cells, we sorted CD4^+^CD8^-^CD25^+^CD44^+^, CD4^+^CD8^-^CD25^+^CD44^-^, CD4^+^CD8^-^CD25^-^CD44^+^, and CD4^+^CD8^-^CD25^-^CD44^- ^cells from the thymus and measured the levels of IL-10 by real-time RT-PCR. It was noted in Figure 5F that among these four CD4^+ ^cell subsets, CD25^+^CD44^+ ^cells expressed the highest levels of IL-10 mRNA, and therefore represented the T_reg _cell population with the highest suppressive activity. There were no major differences with regard to IL-10 expression for CD25^+^CD44^- ^and CD25^-^CD44^+ ^cells. Thus, CD4^+^CD25^+^CD44^+ ^and CD4^+^CD25^+^CD44^- ^cells both displayed regulatory functions, but the former displayed more potent T_reg _activity than the latter.

### CD44 T_reg _cells produce IL-10 that suppresses T cell proliferation

**Figure 6:**
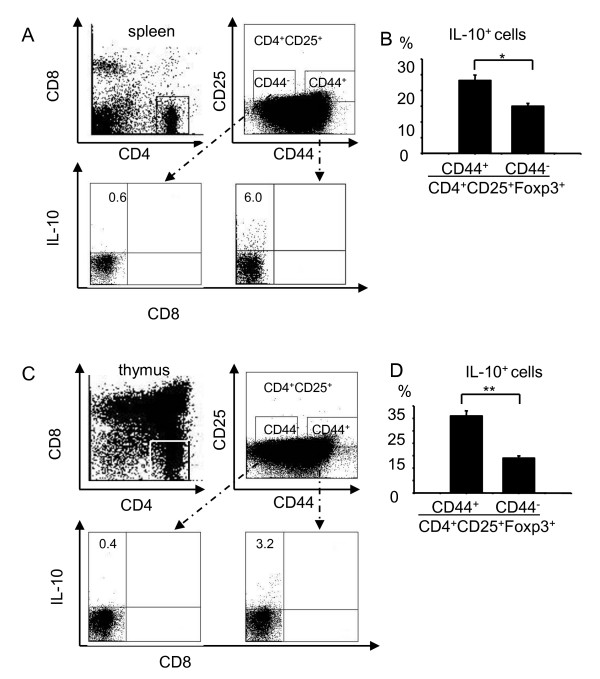
**Intracelluler IL-10 levels in T_reg _cells of thymocytes and splenocytes**. Splenocytes **(A and B) **and thymocytes **(C and D) **were obtained from naïve C57BL/6 mice, stained with mAbs against CD4 (PE-Cy7), CD8 (FITC), CD44 (PE), CD25 (APC-Alexa Fluor755), Foxp3 (APC) and IL-10 (PerCP), and analyzed by flow cytometry. **(A and C) **The percentages of IL-10^+ ^cells in CD4^+^CD25^+^CD44^- ^and CD4^+^CD25^+^CD44^+ ^cells. **(B and D) **The percentages of IL-10^+ ^cells in CD4^+^CD25^+^CD44^-^FoxP3^+ ^and CD4^+^CD25^+^CD44^+ ^FoxP3^+ ^cells. Shown are representative results from one of four independent experiments. Data in B and D are presented as mean ± S.D.

To determine that IL-10 was produced by Foxp3^+ ^T cells but not by other cells, we isolated thymocytes and splenocytes from naïve mice and stained them with antibodies against CD4, CD8, CD25, CD44, IL-10. As shown in Figure 6A and 6C, we found that a significantly higher proportion of CD4^+^CD25^+^CD44^+ ^cells produced IL-10 in the splenocytes and thymocytes (6.0% and 3.2%, respectively) as compared to CD4^+^CD25^+^CD44^- ^cells in splenocytes and thymocytes (0.6% and 0.4%, respectively, *p *< 0.01). Likewise, the percentages of IL-10^+ ^cells in CD4^+^CD25^+^CD44^+ ^Foxp3^+ ^T cells derived from the spleen and thymus (27% and 31%, respectively) were signficnatly higher than those in CD4^+^CD25^+^CD44^- ^Foxp3^+ ^cells (18% and 15%, respectively, Figure 6B and 6D).

**Figure 7:**
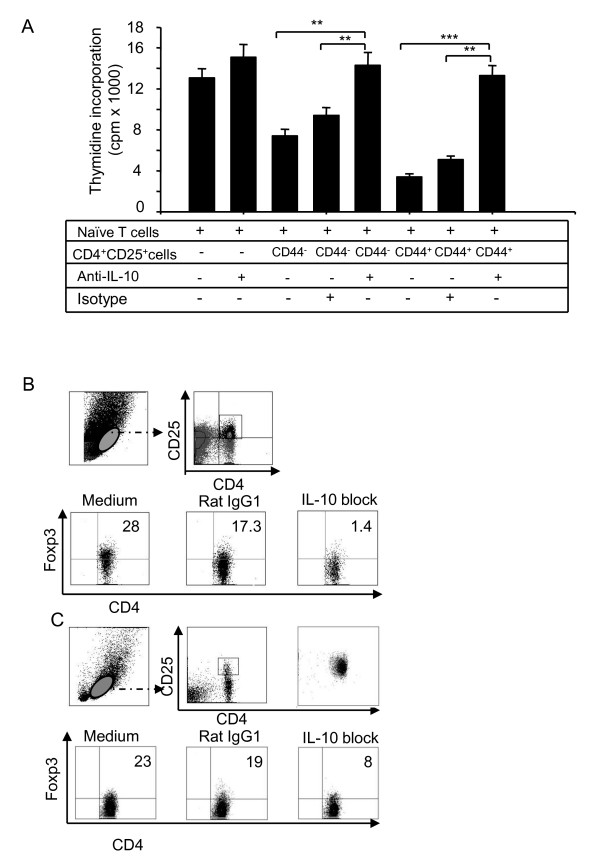
**IL-10 neutralizing antibody reversed suppressive activities of T_reg _cells**. **(A) **CD4^+^CD8^-^CD25^+^CD44^+ ^and CD4^+^CD8^- ^CD25^+^CD44^- ^cells were sorted from splenocytes from C57BL/6 mice and cultured with naïve CD4^+^CD25^- ^T cells in the presence of anti-CD3/anti-CD28 for 72 h. In some cases, anti-IL-10 or isotype control Ab was added. Cell proliferation was measured by [^3^H]-thymidine incorporation. Data are presented as counts for min, and shown are the mean ± S.D. from three independent experiments (** *p *< 0.01, *** *p *< 0.001). **(B) **Splenocytes from naïve mice were treated with either the IL-10 neutralizing antibodies or an isotype control (rat IgG1) in the presence or absence of Con A (5 μg/ml) for 24 h. Cells were stained with antibodies against CD4, CD8, CD25, CD44, and Foxp3 and analyzed by flow cytometry. **(C) **CD4^+^CD25^high ^cells were sorted from splenocytes of C57BL/6 mice and cultured with anti-IL-10 or isotype control Abs for 24 h. Cells were stained with antibodies against Foxp3 and CD4 and analyzed by flow cytometry. Data are presented as the percentage of total splenocytes and shown are representative results of one of four independent experiments.

IL-10 plays a pivotal role in maintaining homestasis *via *direct or indirect control of activation, proliferation, but also *via *its effects on regulatory T cells [25]. To further confirm whether blocking IL-10 aborogated suppressive mechanisms of CD4 T_reg _cells, CD4^+^CD25^- ^and CD4^+^CD25^+^CD44^+^(or CD4^+^CD25^+^CD44^-^) cells were sorted by FACSAria and were incubated alone or co-cultured (CD4^+^CD25^-^: CD4^+ ^CD25^+^) at a 2:1 ratio in the presence of anti-CD3 antibody. In some experiments, blocking antibodies to IL-10 or isotype rat IgG1 control were also used, and after 72 h of incubation, incorporation of radioactivity was measured by liquid scintillation counting in triplicate. As shown in Figure 7A, the suppressive activity of T_reg _cells was aborated when an anti-IL-10 antibody was used.

It is known that Foxp3 controls T_reg _cell development and function. To validate the contribution of IL-10 in regulating T cell development and function, we obtained splenocytes from naïve mice and treated them with an IL-10 neutralizing antibody or an isotype control (rat IgG1) in the presence or absence of Con A (5 μg/ml) for 24 h, with GolgiPlug in the medium for the last 3 h. Cells were stained with antibodies against CD4, CD8, CD25, CD44, and Foxp3 and analyzed by FACS. As shown in Figure 7B, Foxp3 expression levels were much lower (1.4%) in CD4^+^CD25^+^cells treated with anti-IL-10 antibody than those treated with an isotype control (17.3%) or without antibody (28%).

Next, we sorted CD4^+^CD25^high ^cells and incubated them alone or togather with IL-10 blocking antibody or isotype control. After 24 h of incubation, the cells were harvested and anayzed. As shown in Figure 7C, Foxp3 expression were signficnatly decreased by the addition of anti-IL-10 in the CD4^+^CD25^+ ^population of the cells (8%) than those treated with an isotype control (19%) or without antibody (23%). These data suggested an invovlment of IL-10 in suppressive mechanisms of T_reg _cells.

## Discussion

In this study, we have provided evidnece that CD4^+^CD25^+^CD44^+^T_reg _cells expressed high levels of Foxp3, IL-10, and displayed a potent suppressive function *in vitro*. Our results are consistant with those reported by Bookimin *et al*. [26] and Bollyky *et al*. [27], suggesting that CD4^+^CD25^+^CD44^high ^cells display more potent T_reg _functions than do CD4^+^CD25^+^CD44^low ^cells. As expected, these T_reg _cells suppress effector T cell proliferation *via *the production of IL-10. The significance of this study is that it highlights the functional relevence of CD44 in defining T_reg _cell subsets and may explain the unique developmental pathway of CD4^+^T_reg _cells in the mouse thymus and the subtle differences between various T_reg _cell subsets.

The use of better markers or marker combinations in multi-color flow cytometry has made it possible to resolve and define many very small populations of lymphoid progenitors and mature cells. For example, Seddiki *et al*. used 38 surface markers and revealed the persistence of naïve CD45RA^+ ^T_reg _cells in infant thymus, cord or adult peripheral blood, lymph nodes, and spleen [28]. In this study, the simultaneous use of mAbs specific for CD4, CD8, CD25 and CD44 allowed us to detect 16 subtypes of thymocytes, with 80% of the thymocytes as being CD4^+^CD8^+ ^cells (data no shown). Of note, CD4^+^CD8^-^CD25^+^CD44^+ ^and CD4^+^CD8^-^CD25^+^CD44^- ^T_reg _cells in the thymus constituted only 0.4% and 0.8%, respectively, of the total thymocytes (Figure 1B). While 72% of CD4^+^CD8^-^CD25^+^CD44^+ ^cells expressed Foxp3, only 28% of CD4^+^CD8^-^CD25^+^CD44^- ^cells expressed Foxp3 (Figure 1C).

The TCR-β locus plays an important role in the development of T cells [23,24]. Although in TCR transgenic mice, exposure of the developing T cells to the cognate peptide in the thymus causes an increase in the CD4^+^CD25^+ ^T_reg _cell population [3,29], it is unclear how the TCRβ-chain regulates T_reg _cells. We examined TCR-β expression in thymic T_reg _cell development. Cells in these transition stages begin to express TCR genes. We found that CD4^+^CD8^-^CD25^+^CD44^+ ^cells expressed higher levels of TCR-β than did CD4^+^CD8^-^CD25^+^CD44^- ^cells (Figure 2B), and that CD44^high ^T_reg _cells from the thymus displayed higher levels of TCR-β than did CD44^med ^or CD44^low ^cells. Almost 100% of CD44^high ^T_reg _cells expressed the TCR-β-chain, whereas the frequency of TCR-β-expressing cells was significantly lower in T_reg _cells with reduced CD44 expression (Figure 2C). Thus, our data suggest that the levels of TCR-β expression indicate the maturation status of T_reg _cells and correlate with the suppressive function of T_reg _cells. It is possible that CD4^+^CD25^+ ^T cells require activation *via *their TCR to differentiate into suppressive cells [30]. Here, it should be mentioned that at the CD4 SP stage, thymocytes have already passed positive selection by virtue of TCR mediated signaling events. Nevertheless, our data implies that ~60% of CD4 SP CD25^+^CD44^- ^cells do not express a TCR-β chain. It is not clear whether TCR-β^- ^cells might reflect a "spillover" from the double negative (DN) compartment and this possibility will be examined in the future.

It is known that Foxp3 is a conserved transcription factor that programs the development and the suppressive function of CD4^+^CD25^+ ^T_reg _cells. However, less is known about the Foxp3 expression pattern during thymocyte development. To address this issue, we examined the inter-relationship among the expression profiles of CD25, Foxp3, and CD44 and found several trends (Figure 3). Since CD4^+^CD8^-^CD44^+ ^SP or CD4^+^CD8^-^CD44^high ^SP cells expressed high levels of Foxp3, our data suggest a positive correlation between Foxp3 expression and expression of CD44 and the TCR-β-chain. Additional precursor progeny studies are needed to further test/validate this hypothesis.

Because functional assays are critical parameters for assessing T_reg _cell function, we used CD4, CD8, CD25, and CD44 markers and the FACSAria sorter to carefully isolate T cell subsets from the spleens of naïve mice. *In vitro *T cell co-culture and RT-PCR assays indicated stronger suppressive function and higher mRNA levels of IL-10 in CD4^+^CD8^-^CD25^+^CD44^+ ^cells than in CD4^+^CD8^-^CD25^+^CD44^- ^cells (Figure 5). Because CD4^+^CD8^-^CD25^+^CD44^high ^cells displayed the highest suppressive function and the highest mRNA levels of IL-10 (Figure 5E), we suggest that CD44 can be considered a complementary marker for the functional potential of T_reg _cells. To further support this conclusion, we isolated four subsets of CD4^+ ^thymocytes and examined their IL-10 mRNA levels *via *real-time RT-PCR analysis. We provided two lines of evidence indicating the role of IL-10 in T_reg _cell-mediated suppression. First, CD4^+^CD25^+^CD44^+ ^Foxp3^+^cells expressed much higher levels of intracellular IL-10 than did CD4^+^CD25^+^CD44^- ^Foxp3^+ ^cells (Figure 6). Second, the addition of an IL-10 neutralizing antibody reversed this suppression (Figure 7A), and affected Foxp3 expression in the splenocytes (Figures 7B and 7C). Whether, IL-10 neutralizing antibody reversed this suppression through regulating Foxp3 expression is currently not known and will be explored in the future. One possibility exists that our cell population might contain IL-10 producing non-Foxp3^+ ^T cells, so called Tr1-like cells, that could cause reduction in T_reg _cell suppressive effect using anti-IL-10 antibodies; however, this possibility seemed highly unlikely but will be examined in our future studies.

Our results suggest that the regulatory/suppressive potential of these cells can be ranked in the following order: CD4^+^CD8^-^CD25^+^CD44^+ ^cells > CD4^+^CD8^-^CD25^+^CD44^- ^cells > CD4^+^CD8^-^CD25^-^CD44^+ ^cells. Thus, one would predict that naïve CD4^+ ^T cells may have a higher possibility to convert into T_reg _cells in the periphery than do their counterparts. Additional experiments are warranted to test this possibility, because a better understanding of T_reg _cell conversion and acquisition has potential therapeutic utility for autoimmunity and transplantation.

In summary, we found two novel phenotypes of T_reg _cells in the thymus. While both CD4^+^CD25^+^CD44^+ ^and CD4^+^CD25^+^CD44^- ^cells display suppressive activities, CD4^+^CD25^+^CD44^high ^cells are the most potent T_reg _cells. The expression levels of CD44 positively correlate with the expression of IL-10 and Foxp3, as well as with the regulatory potential of T_reg _cells which suppresses T cell proliferation function by producing IL-10.

## Competing interests

The authors declare that they have no competing interests.

## Authors' contributions

TL and LS contributed equally to the design of the study and writing of the manuscript; TL performed the experiments and analyzed data; GL and RK participated in experimental design, data analysis, and manuscript preparation; AKC provided support for this research, contributed in designing the study and manuscript writing and revisions, as well as in responding to reviewers' comments. All authors substantially contributed to the redaction of the manuscript and have given final approval on the version to be published.

## Reviewer's Comments

### Reviewers' report 1

Dr. Lenardo

Liu et al., demonstrate CD44 as a new marker for Tregs in their manuscript entitled "The development of CD4^+^CD25^+^CD44^+ ^regulatory T cells in mouse thymus". The authors have clearly shown that Foxp3 expression and enhanced suppressive activity of Tregs are associated with CD44 molecule. The authors have presented a concise report of their findings in a well-written manuscript and clearly presented data. They have also speculated that Tregs may develop from DN2 or DN3 stage T cells separately before development of naïve cells.

*Authors' response*: *We were very pleased to read your supporting comments on our study and felt that your suggestions were very helpful in assisting us to improve the quality of the revised manuscript. Based upon your suggestions, we have performed new experiments and presented these data in new Figures *6*and *7. *In addition, we have addressed your specific comments point-by-point*.

**1) **The authors have not shown any evidence with regards to the Treg development other than CD25 and CD44 staining. Therefore, the title is misleading and changes the focus of the actual content of the paper i.e., CD44 correlates with Foxp3^+ ^expression. There is no scientific evidence presented in the paper, showing that Treg cells actually arise from DN3 or 2/DN3 cell stage cells. The possibility that Treg cells could develop from DN4 cell stage cells by reacquiring CD44 and CD25 during negative selection and maturation processes has not been formally excluded. There is a school of thought that Treg cells escape negative selection even-though they have high affinity self-ligands. The authors should therefore address these points if they want to discuss about developmental aspects of Tregs in this paper. Otherwise, the authors should change the title of the manuscript and speculate the developmental aspects of Tregs only in the "discussion" section.

*Authors' response*: *To address question 1, we have changed the title to "CD44 expression positively correlates with Foxp3 expression and suppressive function of CD4*^+^*T*_*reg *_*cells", as suggested by the reviewer. We have also removed the original Figure Seven from the revised manuscript and provided a brief discussion on the development of T*_*reg *_*cells in the revised discussion of the manuscript*.

**2) **It is interesting that CD44 expression is coupled to the TGF-β and IL-10 expression. However, it has been shown previously that TGF-β and IL-10 are not necessary for *in vitro *suppression. Therefore, if authors claim CD44 expression positively correlates with higher suppression and these suppressive cytokines, the authors should demonstrate if CD44 high subset of Tregs suppress predominantly by these suppressive cytokines. It would be interesting to test whether the suppressive activity of CD44^+ ^subset is blocked by neutralizing these cytokines. It is possible that CD44^- ^subset and CD44^+ ^subset of CD25^+ ^Tregs suppress in different manner, which is why one cannot abrogate suppression by neutralizing TGF-β and IL-10 in cultures with both the subsets so far.

*Authors' response: To address question 2, our new data showed that suppressive function of CD4 T*_*reg *_*cells on T cell proliferation could be reversed by neutralizing anti-IL-10 antibodies (Figure *7A). *Further, our data provided evidence that IL-10 might regulate suppression of T cell proliferation function by modulating Foxp3 (Figure *7B).

### Reviewers' report 2

Dr Klein & Dr Wirnsberger (nominated by Dr JC Zungia-Pfluker)

The manuscript "CD44 expression positively correlates with Foxp3 expression and suppressive function of CD4^+ ^Treg cells" by Liu et al. proposes a subdivision of thymic and splenic Treg into populations characterized by different levels of CD44 expression. Although the revised ms deals with the functional properties of these subpopulations rather than focusing on developmental aspects of Treg cell biology, several major concerns remain as to the characterization of these cells and the interpretation of the reported experiments. Because the focus of the ms has profoundly changed, we have looked at it as if it were a first submission.

**1) **Introduction: *"...the surface expression of CD44 in mouse thymocytes preceded that of Foxp3" - *this statement implies a direct precursor/progeny relationship between the described subpopulations and "mature" Treg that is not experimentally addressed at all in this manuscript.

*Authors' response: Since our data indicated that CD44 expression positively correlated with Foxp3 expression, we have revised this line in the introduction to correctly reflect our data*.

**2) **Figure 1: The authors claim that "...*location and sequence of Treg development remain unclear." *Significant progress has been made in terms of a delineation of critical events during the earliest phase(s) of Treg differentiation and also in terms of the localization of their differentiation, which are not referenced here (Lio et al., 2008, Lee et al., 2009, Fontenot et al., 2005).

The following characterization of thymocyte subpopulations is based upon CD4/8/25/44 staining. Although the authors avoid calling these populations Treg at that point, the subsequent experiments are done using this staining scheme and cells are referred to as "*thymic Treg cells*" later on. The only specific Treg marker to date is Foxp3, hence - especially given the already reported heterogeneity of CD4 SP CD25^+ ^cells - Foxp3 staining would be essential for these types of analyses.

*Authors' response: As suggested by the reviewer, we have added the following references: Lio et al., 2008, Lee et al., 2009, Fontenot et al., 2005 in the revised manuscript. In addition, we have performed new experiments and added data showing "Foxp3 expression in CD4*^+^*CD8*^-^*CD25*^+^*CD44*^+^*and CD4*^+^*CD8*^-^*CD25*^+^*CD44*^-^*cells (see new Figure *1C).

**3) **Figure 2: At the CD4 SP stage, thymocytes have already passed positive selection by virtue of TCR mediated signaling events. Additionally, as stated in the introduction, Treg differentiation is thought to rely on thymic antigen encounter/TCR signaling. Nevertheless, Figure 2 implies that ~60% of CD4 SP CD25^+^CD44^- ^("Treg") cells do not express a TCR-β chain. This discrepancy is not discussed in the manuscript at all and might reflect a "spillover" from the DN compartment.

*Authors' response: We agree with the reviewer that our original Figure *2*was not very clear and that it may cause some confusion. The revised Figure *2*now only focuses on TCR-β expression among CD4*^+^*CD25*^+^*CD44*^-^*and CD4*^+^*CD25*^+^*CD44*^+^*SP cells. We have expanded the discussion section and included reviewer's point in this section*.

**4) **Figure 3: The authors also claim to detect both different percentages of TCR-β^+ ^cells and different levels of TCR-β expression among the described populations. Staining showing different levels of TCR-β expression among the described subsets are not provided, however. The suggestion that *"...CD4*^+^*CD25*^+^*CD44*^+^*cells represent a more mature subset of Treg cells..." *based upon the presented staining (lacking Foxp3 staining) and lacking any experiments providing evidence for a precursor/progeny relationship is daring.

*Authors' response: We think that the reviewer meant Figure *2*and not Figure *3. *We found that CD4*^+^*CD25*^+^*CD44*^+^*cells expressed the highest level TCR-β in thymocytes. So we believe that CD4*^+^*CD25*^+^*CD44*^+^*cells were more mature T cells. We meant mature T cells and not Treg cells*.

**5) **Data in Figure 3 show that essentially all Foxp3^+ ^cells are also CD44^+^/high, but that only a "small" fraction of CD4^+^CD25^+^CD44^+ ^cells - as classified by the authors - are Foxp3^+^. These results clearly necessitate a re-examination of the results and interpretation of Figure 1 and *2*. The results presented also argue for a relatively low percentage of CD25^+^/high cells being Foxp3^+^, which is not consistent with the literature on Foxp3^+ ^cells in the (adult) thymus (Fontenot et al., 2005).

*Authors' response: We appreciate for reviewer's insightful comments. In more than 10 independent repeats, we sometimes detected higher frequencies and sometimes lower frequencies of Foxp3*^+^*cells. To confirm the trends, we carefully performed additional experiments. After careful reviewing all of our data, especially the new data, we decided to present a revised Figure *3, *showing our new data. This is not to say that the old data were wrong, since it is a cellular staining, Foxp3 expression varied in different samples. Importantly, however, the rate of Foxp3 expression in CD44*^+^*and CD44*^-^*subset was similar in all of the data analyzed*.

**6) **Figure 4: The authors claim to have done a *"functional assessment of Treg cell subsets in mouse thymus and spleen." *The data presented only show the suppressive activity of splenocytes. (For thymocytes: only Real Time PCR data are provided). The differences in suppressive potency (Figure 4A) are very modest. Additionally the authors show, that the described populations differ in their TGF-beta and IL-10 mRNA expression levels. The differences are modest and probably not too informative, however.

*Authors' response: The only ways to show "functional assessment of Treg cell subsets in mouse thymus and spleen" are by RT-PCR, real Time RT-PCR and intercellular staining, and we obtained similar results with these different assays. Our experiments were limited by cell sorting conditions, as we could not sort enough of CD44 Treg cells, which influenced our results. However, we have provided new data in which Treg: effector cells were used in a ratio of 1:2, and the suppressive potency was impressive (new Figure *5A).

**7) **Figures 4 and 5 are redundant. It might not be necessary to show Figure 4 at all. RT PCR (Figure 5) and Real-time data (Figure 6A) are redundant. Assessing cytokine mRNA expression levels does not allow any statements on the suppressive potential of thymocyte populations (6B).

*Authors' response: We agree that the original Figures *4*and *5*are redundant and have deleted Figure *5. *We feel that Figure *6B*can help readers understand cytokine expression of Treg cells in the thymus, and have decided to have new Figures *5E*and *5F.

**8) **Figure 7: The authors claim that *"CD44 Treg cells produce IL-10 and TGF-beta cytokines that suppress T cell proliferation"*. The expression of TGF-beta by the described populations is not shown, however. Data on a role for TGF-beta in *in vitro *suppression assays is also not provided.

*Authors' response: We could not detect TGF-beta expression in both CD44*^+^*and CD44*^- ^*cells with flow cytometry assay and the TGF-beta mRNA data were modest. We decided to delete TGF-beta data and to focus on IL-10 in Treg cells in later experiments*.

**9) **Figure 8B: involvement of IL-10 in direct regulation of Foxp3 expression seems to be problematic. Studies on a role of IL-10 and TGF-beta in Treg cell induction/homeostasis and suppressor function (Li and Flavell 2008, amongst many others) and a possible role for CD44 and Foxp3^+ ^cells can - via intracellular staining - be visualized as a distinct population among CD4^+ ^cells. The CD4/Foxp3 plots in Figure 8B do not really allow for gating on Foxp3^+ ^cells. Due to the experimental setup and the lack of "resolution" provided by the Foxp3 staining shown an interpretation suggesting an low/high molecular weight hyaluronic-acid in Treg function (Bollyky et al., 2007) have been reported elsewhere and are not sufficiently referenced in this section.

In aggregate, this manuscript provides some insights into how Treg can be subdivided into subpopulations differing in their suppressive potency based upon the expression of the hyaluronic-acid receptor CD44. However, the concerns mentioned above should be addressed in order to clarify the validity of the given interpretations and conclusions. We hope that these comments are helpful to improve the quality of this manuscript.

*Authors' response: In this study, we have used anti-IL-10 antibody to block IL-10 in splenocytes, and we found that IL-10 induced Foxp3 expression was decreased. Furthermore, we sorted CD4CD25*^high^*cells from spleen of C57BL/6 mice, cultured themwith anti-IL-10 or rat IgG1 antibody for 24 hours, and analyzed data by Flow cytometry (revised Figure *7C). *We found IL-10 induced Foxp3 expression was decreased in CD4CD25*^high^*cells after blocking this cytokine with IL-10 neutralizing antibody. These results suggest that Treg cell suppressive effect on T cell proliferation could be reversed by IL-10 blocking antibody through regulation of the Foxp3 expression*.

Finally, we have added Li, Ming O and Richard A Flavell 2008 reference and that of Bollyky et al. 2007 in the revised manuscript

### Reviewers' report 3

Dr Shevach

The authors have changed the title of the paper and this does improve the focus of the manuscript. However, numerous issues remain to be resolved with this manuscript:

**1) **The authors now accept that Foxp3 is the marker for Treg in the mouse, yet the data presented in figures 1, 2, 3 add absolutely nothing to our understanding of the development of Foxp3^+ ^T cells in the thymus. Although they appear to perform competent staining, the level of Foxp3 expressing cells even in the CD44^high ^pool is only 20%. It is not clear what new information is conveyed in these figures.

*Authors' response: Although much is known about T cell development in the thymus, there is limited information on T*_*reg *_*cell markers in the thymus. Figure *1*showsCD4*^+^*CD8*^-^*CD25*^+^*CD44*^+^*and CD4*^+^*CD8*^-^*CD25*^+^*CD44*^-^*cells in thymus, and Foxp3 expression in CD4*^+^*CD8*^-^*CD25*^+^*CD44*^+^*and CD4*^+^*CD8*^-^*CD25*^+^*CD44*^-^*cells. Figure *2*shows that TCR-β locus plays an important role in the development of T cells. We compared the expression of TCR-β in both CD4*^+^*CD25*^+^*CD44*^+^*and CD4*^+^*CD25*^+^*CD44*^- ^*cells, and found that CD4*^+^*CD25*^+^*CD44*^+^*cells are mature cells. Our data in Figure *3*show that Foxp3 is positively correlated with CD44. Based on our new data, we have revised Figure *3.

**2) **The authors need to quantitate by intracellular staining the percentage of Foxp3 expressing cells in each of their so-called Treg populations in figure 4. As pointed out in my previous review, the magnitude of suppression in this figure is not great (compared to other published studies) and the differences between the CD44^+ ^and the CD44^-^populations could easily be accounted by minor differences in percentage of Foxp3^+ ^T cells. The same criticism applies to the data in figure 5.

*Authors' response: We have quantitated by intracellular staining, the percentage of Foxp3 expressing cells in splenocytes (Figure *4). *Our experiments were limited by cell sorting conditions, as we could not get sufficient numbers of CD44 T*_*reg *_*cells. However, we have provided new data in which Treg: effector cells were used in a ratio of 1:2, and the suppressive potency was impressive (new Figure *5A).

**3) **The authors rely heavily on PCR data in figure 6. As suppression requires TCR activation, they need to stimulate the populations to determine how much of these cytokines they are capable of producing. Elisa assays and intracellular staining are needed. MOST IMPORTANTLY, they need to do simultaneous staining for IL-10 and Foxp3. We agree that this it is difficult to analyze TGF-beta by IC staining.

*Authors' response: We have performed this study in Figure *6. *The most critical step for detection of intracellular accumulation of cytokines by intracellular staining is activation of a cell population to induce production of cytokines of interest. We used Con A as an activator to co-culture with GolgiPlug in the medium. There was no IL-10 expression in control cells which were not activated by Con A*.

**4**) The same criticism holds for the data in figure 7. Are the cells that stain for IL-10 Foxp3^+^? CD44 and CD25 are surrogate markers that mean little.

*Authors' response: Yes, we have added data on IL-10 levels in CD4*^+^*CD8*^-^*CD25*^+^*CD44*^+^*Foxp3*^+^*and CD4*^+^*CD8*^-^*CD25*^+^*CD44*^-^*Foxp3*^+^*cells in the revised Figures *6B*and *6D.

**5) **The suppression data in figure 8 lacks an important control. The authors need to add the anti-IL-10 to the naïve T cells alone. Anti-IL-10 will frequently increase the response of this population as well. In general, anti-CD28 reverses suppression in the mouse model and it is very difficult to suppress mouse T cell activation in the presence of anti-CD28 (the authors might review some of the papers published years ago which address this point). It is unclear why the authors see a reversal of suppression with anti-IL-10 as other groups using highly purified Foxp3^+ ^T cells have not seen this. There seems to be no difference in the susceptibility of the high versus the low population to anti-IL-10 reversal, so one would assume that their data should not differ from the published data using Tregs that are not fractionated based on CD44 expression. One possibility is that the cell populations contain IL-10 producing non-Foxp3^+ ^T cells, so called Tr1-like cells.

*Authors' response: Anti-IL-10 with naïve T cell data have been added (only anti-CD3 and not anti-CD28 in the medium) as a control. We have sorted CD4CD25*^high^*cells from spleen of C57BL/6 mice and cultured them with anti-IL-10 or Rat IgG1 for 24 hours, and analyzed by Flow cytometry (revised Figure *7C). *Our data clearly and very reproducibly indicated that anti-IL-10 antibodies reversed the suppressive ability of T*_*reg *_*cells. Therefore, we are convinced that our data is accurate. However, it is possible that the Foxp3*^+^*cells isolated from transgenic mice might behave differently compared to Foxp3*^+ ^*cells from normal mice. The reviewer brought up an interesting point regarding Tr1-like cells. We have added this statement in the revised manuscript to cover all points*.

**6) **a) Panel B of figure 8 is simply fantasy. I see no physiologic relevance to studying an unseparated population of thymocytes and splenocytes. b) Why do the authors add Con A? IL-10 deficient mice have normal numbers of Foxp3^+ ^T cells. c) Why is there a difference between the medium control and the control IgG? d) This study needs to be performed with purified Foxp3^+ ^cells from thymus and spleen. Considerable cell death occurs when thymocytes are cultured under these conditions, yet cell survival is not mentioned.

*Authors' response***: *a) ****In this study, we have used anti-IL-10 to block IL-10 in splenocytes and found that IL-10 induced Foxp3 expression was decreased. Furthermore, we sorted CD4CD25*^high^*cells from spleen of C57BL/6 mice and cultured them with anti-IL-10 or Rat IgG1 for 24 hours, and analyzed samples by Flow cytometry (revised Figure *7C). *We found IL-10 induced Foxp3 expression was decreased in CD4CD25*^high^*cells*. ***b) ****In panel B of Figure *7, *our data showed different level of Foxp3 expression between anti-IL-10 treated-versus control-cells and medium alone. However, more significant difference in Foxp3 expression between anti-IL-10 treated- and control-cells in the presence of ConA was noted. ConA may increase activity of T cells to influence Foxp3 expression on T*_reg _*cells. However, we did not use ConA in our new Figure *7C. ***c) ****We believe that this antibody might not be highly purified; however, it did not influence the accuracy of our results*. ***d) ****Spleen samples but not thymus were used and the cells did not die in these experiments*.
